# A randomized controlled study evaluating safety and efficacy of leuprorelin acetate every-3-months depot for 2 versus 3 or more years with tamoxifen for 5 years as adjuvant treatment in premenopausal patients with endocrine-responsive breast cancer

**DOI:** 10.1007/s12282-015-0593-z

**Published:** 2015-02-06

**Authors:** Eiichi Shiba, Hiroko Yamashita, Junichi Kurebayashi, Shinzaburo Noguchi, Hirotaka Iwase, Yasuo Ohashi, Kiyofumi Sasai, Tsukasa Fujimoto

**Affiliations:** 1 Department of Breast Surgery, Osaka Breast Clinic, 1-3-4 Fukushima, Fukushima-ku, Osaka, 553-0003 Japan; 2 Department of Breast and Endocrine Surgery, Nagoya City University Hospital, Nagoya, Japan; 3 Present Address: Department of Breast Surgery, Hokkaido University Hospital, Sapporo, Japan; 4 Department of Breast and Thyroid Surgery, Kawasaki Medical School, Kurashiki, Japan; 5 Department of Breast and Endocrine Surgery, Osaka University Graduate School of Medicine, Osaka, Japan; 6 Department of Breast and Endocrine Surgery, Kumamoto University, Kumamoto, Japan; 7 Department of Integrated Science and Engineering for Sustainable Society, Chuo University, Tokyo, Japan; 8 Takeda Pharmaceutical Company Limited, Osaka, Japan

**Keywords:** Premenopausal endocrine-responsive breast cancer, Adjuvant endocrine therapy, Leuprorelin acetate, Safety, Disease-free survival (DFS)

## Abstract

**Background:**

Luteinizing hormone-releasing hormone (LH-RH) agonists provide effective adjuvant treatment for premenopausal women with endocrine-responsive breast cancer. Here, we investigated appropriate treatment durations of an LH-RH agonist, leuprorelin.

**Methods:**

We conducted an open-label, randomized controlled pilot study to evaluate the safety and efficacy of leuprorelin subcutaneously administered every-3-months for 2 versus 3 or more, up to 5 years, together with daily tamoxifen for 5 years in premenopausal endocrine-responsive breast cancer patients. Primary endpoints were disease-free survival (DFS) and safety.

**Results:**

Eligible patients (*N* = 222) were randomly assigned to receive leuprorelin for either 2 years (*N* = 112) or 3 or more years (*N* = 110) with tamoxifen for 5 years after surgery. Leuprorelin treatment for 3 or more years provided no significant difference in DFS rate over 2 years: 94.1 versus 91.8 % at 144 weeks (3 years) after the second year (week 96) and 90.8 versus 90.4 % at the fifth year (week 240). The overall survival rate was 100 % for both groups during the third through fifth year study period. There were no significant differences in the incidence of adverse events (AEs) between the 2 groups: most AEs were rated grade 1 or 2.

**Conclusions:**

Adjuvant leuprorelin treatment for 3 or more years with tamoxifen showed a survival benefit and safety profile similar to that for 2 years in premenopausal endocrine-responsive breast cancer patients. No new safety signal was identified for long-term leuprorelin treatment. Longer follow-up observation is needed to determine the optimal duration of leuprorelin treatment.

## Introduction

Luteinizing hormone-releasing hormone (LH-RH) agonists are effective adjuvant therapy for premenopausal women with endocrine-responsive breast cancer [1–4]. Tamoxifen, the most firmly established adjuvant therapy, has been used as a standard adjuvant therapy for pre- and postmenopausal women with early breast cancer [5–8]; however, it can result in stimulation of pituitary-ovarian function, accompanied by increased serum estradiol (E_2_) levels [9]. A meta-analysis of 4 randomized clinical trials showed that the combination of tamoxifen plus LH-RH agonist was superior to LH-RH agonist alone in providing a significant survival benefit [10–12]. Currently, the combination of 5 years of tamoxifen plus 2 years of ovarian ablation with an LH-RH agonist is frequently used as a postoperative adjuvant therapy for premenopausal women with early breast cancer in many countries. The St Gallen international expert consensus on the primary therapy of early breast cancer has recommended 5 years of tamoxifen alone or in combination with 5 years of ovarian suppression as a standard adjuvant therapy for premenopausal breast cancer patients [13, 14]. Since there have been few studies reporting the clinical outcome with 5 years of LH-RH agonists [15], however, the optimal duration of LH-RH agonists has yet to be elucidated.

Leuprorelin acetate (leuprorelin), an LH-RH agonist, is available as depot formulations for subcutaneous administration every 1- or 3-months. It is used worldwide for the treatment of hormone-responsive cancers, such as prostate cancer [16] and premenopausal breast cancer [17–20], as well as estrogen-dependent conditions such as endometriosis and uterine fibroids. In premenopausal patients with ER-positive, node-positive breast cancer, leuprorelin administered every-3-months depot showed a non-inferior effect to chemotherapy with cyclophosphamide, methotrexate, and fluorouracil (CMF), and was well tolerated [21].

To investigate the appropriate treatment duration for leuprorelin, we conducted an open-label, randomized controlled pilot study evaluating the safety and efficacy of adjuvant therapy with leuprorelin administered every-3-months for 2 years versus 3 or more up to 5 years in combination with tamoxifen given daily for 5 years in premenopausal women with endocrine-responsive breast cancer.

## Patients and methods

### Study design

Eligible patients were randomly assigned at a 1:1 ratio to receive leuprorelin (11.25 mg) subcutaneous administration every-3-months depot either for 2 years or for 3 or more years, up to 5 years, in combination with tamoxifen (20 mg daily) given orally for 5 years. Random assignment was performed using dynamic allocation with the number of positive axillary lymph nodes (0, 1–3, ≥4), tumor diameter (≤2 cm, >2 cm), estrogen receptor (ER)/progesterone receptor (PgR) status (ER+/PgR+, ER+/PgR−, ER−/PgR+), age (at the time of enrollment; ≤39, 40–44, ≥45 years), pre- and post-operative chemotherapy (presence, absence), and study site.

Patients assigned to the 2-year treatment group received leuprorelin every-3-months depot for 2 years (96 weeks), and tamoxifen for 5 years (240 weeks). For the 3-or-more-year treatment group, patients who completed 3 years (144 weeks) of leuprorelin treatment could extend that treatment for up to 5 years (240 weeks in total) if they were considered appropriate for continuing the extension study in consideration of safety etc. and gave written informed consent for it, while tamoxifen was administered throughout the 5-year study period. Patients were also allowed to receive anti-osteoporosis drugs except for zoledronic acid as needed.

This study was conducted in accordance with the International Conference on Harmonisation of Good Clinical Practice Guidelines, the principles of the Declaration of Helsinki, and all applicable laws and regulations at 19 medical centers in Japan between June 2006 and March 2013. The protocol was reviewed and approved by the Institutional Review Boards in all of the participating study sites. All patients provided written informed consent for participation before enrollment in the study.

### Patients

Premenopausal patients with histologically confirmed primary breast cancer who met the following criteria were eligible for this study: age ≥20 years; both or either ER+ or PgR+ primary tumor; T1 to T3, any N, and M0, according to the TNM classification (Union for International Cancer Control, Sixth Edition, 2002); any type of surgical procedure (in the case of breast-conserving surgery, postoperative radiation to the breast was required); any type of preoperative treatment and postoperative adjuvant chemotherapy prior to enrollment; capable of receiving the study drug and tamoxifen within 12 weeks after surgery or after postoperative adjuvant chemotherapy prior to enrollment (postoperative chemotherapy had to be completed by the time of enrollment); history of regular menstruation within 12 weeks prior to enrollment, or follicle-stimulating hormone (FSH) of <40 mIU/mL and E_2_ of ≥10 pg/mL as measured within 12 weeks prior to enrollment; not having a chemical menopause (FSH of <40 mIU/mL and E_2_ of ≥10 pg/mL) within 12 weeks after completion of postoperative adjuvant chemotherapy; performance status of grade 0 or 1.

Exclusion criteria included the following conditions or situations: endocrine therapy prior to surgery; postoperative adjuvant endocrine therapy before enrollment; bilateral oophorectomy and irradiation to bilateral ovaries; inflammatory breast cancer or bilateral breast cancer; multiple cancers or history of carcinoma in other organs.

### Primary and secondary endpoints

Primary endpoints were disease-free survival [DFS: defined as the time from random assignment to disease event (recurrence, second primary cancer, or death)] and safety throughout the 5-year study period. The secondary efficacy endpoint was overall survival (OS: defined as the time from randomization to death) throughout the 5-year study period. If the observation period ended before any disease event occurred, the DFS time was censored. Other measures included menstruation status, quality of life, and levels of E_2_, LH, and FSH.

Safety data were obtained from the findings on clinical signs/symptoms, physical examinations, vital signs, laboratory test results, and bone mineral density (BMD) as measured by dual-energy x-ray absorptiometry. Adverse events (AEs) were recorded throughout the study period and graded according to the National Cancer Institute Common Terminology Criteria for Adverse Events version 3.0.

### Statistical analysis

Since this study was a pilot study, the sample size was determined considering the feasibility: that is, we planned to enroll 220 patients (110 per group).

For the efficacy analysis, 2 types of datasets were defined. The full analysis set (FAS) was defined as data from the patients receiving at least 1 dose of the study drug after randomization. The modified FAS (mFAS) was defined as data from the patients who were included in the FAS and had been examined at the end of the second year of treatment (week 96) and then continued in the study. The safety analysis set (SAS) was defined as data from the patients who received at least 1 dose of the study drug.

As the first primary analysis for the primary endpoint (DFS), the Kaplan–Meier method was used to estimate the DFS distribution for 144 weeks after the second year (week 96) through the fifth year (week 240) study period for each group in the mFAS. The point estimates for each group and the group difference and their 2-sided 95 % CIs following Greenwood’s formula were calculated at 144 weeks after week 96. The logrank test was applied, and the hazard ratio of the 3-or-more- versus 2-year groups and the 95 % CI were estimated by applying the Cox proportional hazard regression model. Furthermore, similar analyses other than the Cox model approach were planned to evaluate survival outcome throughout the 5-year study period for the FAS as the second primary analysis, because the true hazard ratio between weeks 0 and 96 must be 1, which must be different after week 96. A similar analysis approach was planned for the secondary endpoint (OS). For statistical testing, the significance level was set at 0.05 (2-sided). Statistical multiplicity was not adjusted since this was a pilot study.

AEs were summarized in the SAS based on the Medical Dictionary for Regulatory Activities (MedDRA) terminology version 16.0.

## Results

### Patients

**Fig. 1:**
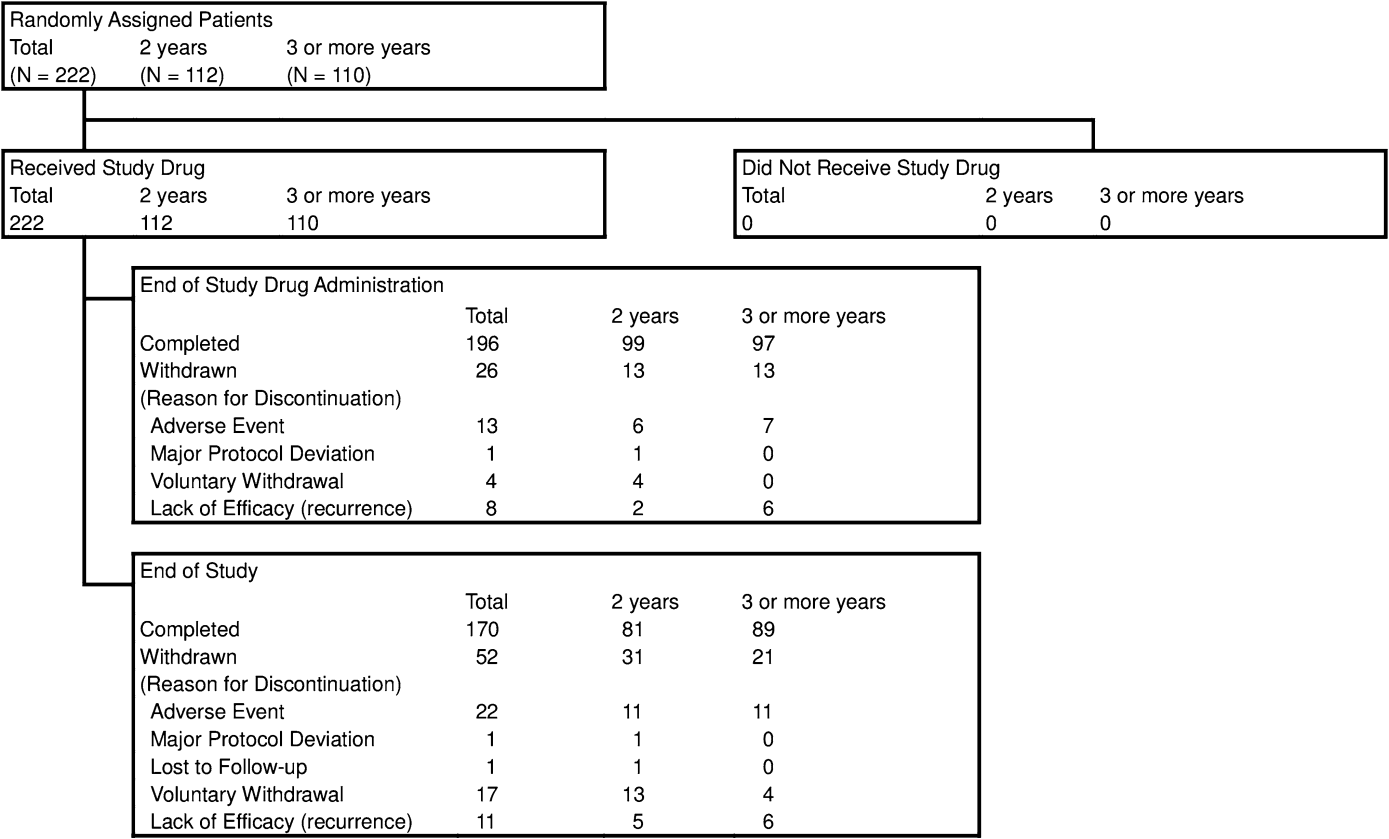
Patient disposition, *N* number of patients evaluated

A total of 222 patients were enrolled between July 2006 and July 2008 and randomly assigned to receive leuprorelin for either 2 years (*N* = 112) or 3 or more years (*N* = 110). Figure 1 shows the patient disposition. Of 222 patients (112 and 110 in the 2- and 3-or-more-year groups, respectively) in the FAS, 196 patients (99 and 97) completed the leuprorelin treatment, and 26 patients (13 and 13) discontinued. Overall, 170 patients (81 and 89) completed the 5-year study period, and 52 patients (31 and 21) discontinued. The mean duration of follow-up was longer in the 3-or-more-year group than in the 2-year group (1555 versus 1459 days), but the median duration (1711 days) in the 3-or-more-year group was comparable to that (1709 days) in the 2-year group.

Table 1 summarizes the baseline demographic and disease characteristics of patients in the FAS. The median age of all patients was 43 years (range 25–56). Tumor stage distribution was I (65.8 %), IIA (27.0 %), IIB (5.0 %), IIIA (1.8 %), and IIIB (0.5 %). The majority of patients were axillary lymph node-negative (89.2 %), and had ER+/PgR+ tumors (93.2 %). Four patients (1.8 %) had preoperative chemotherapy and 23 patients (10.4 %) had postoperative adjuvant chemotherapy. There were no significant differences in baseline characteristics between the groups except for serum E_2_ levels (Wilcoxon *t* test, *p* = 0.033).

**Table 1:** Baseline demographic and disease characteristics of patients

Variable	Overall	Treatment group	
		2 years	3 or more years
	(*N* = 222)	(*N* = 112)	(*N* = 110)
Age (years)			
Median (range)	43.0 (25–56)	43.5 (25–52)	43.0 (27–56)
≤39	65 (29.3)	33 (29.5)	32 (29.1)
40–44	60 (27.0)	28 (25.0)	32 (29.1)
≥45	97 (43.7)	51 (45.5)	46 (41.8)
BMI (kg/m^2^)			
Mean (SD)	21.80 (3.436)	21.79 (3.295)	21.82 (3.589)
Tumor stage (TNM classification)			
I	146 (65.8)	74 (66.1)	72 (65.5)
IIA	60 (27.0)	29 (25.9)	31 (28.2)
IIB	11 (5.0)	6 (5.4)	5 (4.5)
IIIA	4 (1.8)	2 (1.8)	2 (1.8)
IIIB	1 (0.5)	1 (0.9)	0
Tumor size (cm)			
≤2	165 (74.3)	84 (75.0)	81 (73.6)
>2	57 (25.7)	28 (25.0)	29 (26.4)
Number of axillary lymph nodes			
0	198 (89.2)	100 (89.3)	98 (89.1)
1–3	21 (9.5)	10 (8.9)	11 (10.0)
≥4	3 (1.4)	2 (1.8)	1 (0.9)
ER/PgR expression			
ER+/PgR+	207 (93.2)	103 (92.0)	104 (94.5)
ER+/PgR−	9 (4.1)	5 (4.5)	4 (3.6)
ER−/PgR+	6 (2.7)	4 (3.6)	2 (1.8)
Performance status			
0	222 (100)	112 (100)	110 (100)
1–4	0	0	0
Preoperative chemotherapy			
Presence	4 (1.8)	2 (1.8)	2 (1.8)
Absence	218 (98.2)	110 (98.2)	108 (98.2)
Postoperative chemotherapy			
Presence	23 (10.4)	12 (10.7)	11 (10.0)
Absence	199 (89.6)	100 (89.3)	99 (90.0)
Serum estradiol (pg/mL) at week 0			
Median (interquartile range)	90.5 (50.0–170.0)	88.0 (37.0–144.0)	101.5 (59.0–204.0)

Values represent the number (%) of patients unless otherwise indicated

*BMI* body mass index, *SD* standard deviation, *ER* estrogen receptor, *PgR* progesterone receptor

The majority of patients had good medication compliance with the study treatment: 99 (88.4 %) patients in the 2-year group and 97 (88.2 %) patients in the 3-or-more-year group received 8 and 12 doses of leuprorelin specified in the protocol, respectively. Of the 97 patients in the 3-or-more-year group, 5 patients (4.5 %) received 13–19 doses and 76 patients (69.1 %) received 20 doses, the maximum dose in the study. Each group had good compliance with tamoxifen treatment throughout the 5-year study period.

### Outcome

Throughout the 5-year study period, there were 20 disease events (10 each in the 2- and 3-or-more-year groups, respectively): 11 recurrences (5 and 6), 9 second primary cancers (5 and 4). One patient in the 3-or-more-year group who died in a natural disaster (earthquake) was censored at the time of death.

**Fig. 2:**
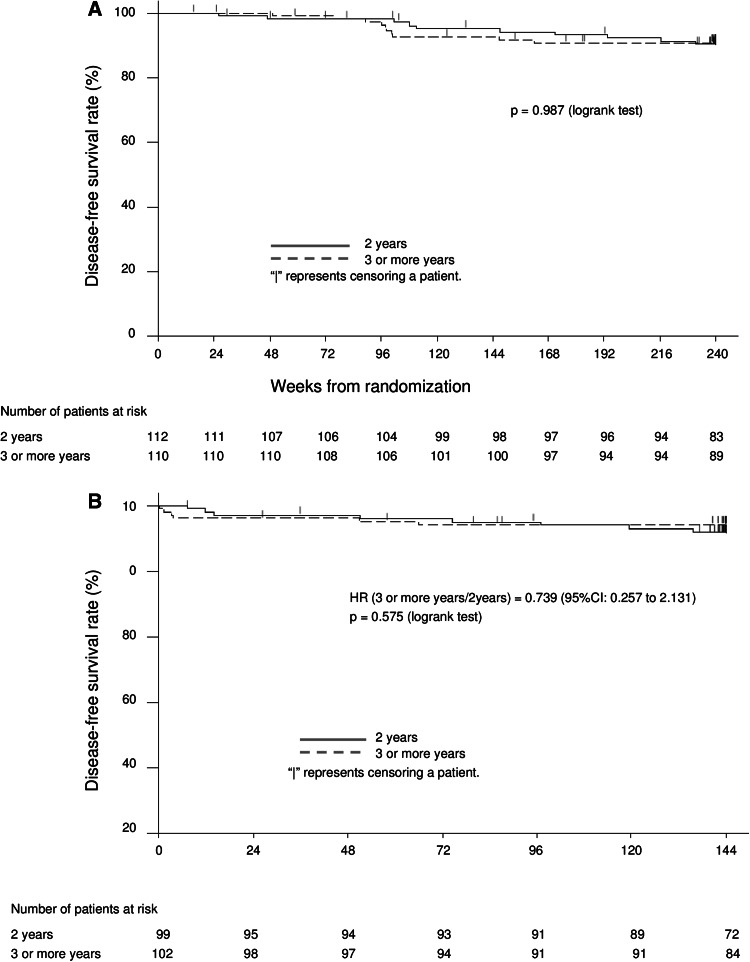
Kaplan–Meier analysis of disease-free survival for patients **a** during the overall 5-year study period and **b** during the third through fifth year study period. *HR* hazard ratio, *CI* confidence interval

Figure 2a shows the Kaplan–Meier analysis of DFS for patients in the FAS. The DFS rate at week 240 was 90.4 % and 90.8 % in the 2- and 3-or-more-year groups, respectively. There were no significant differences between the 2 groups (estimated difference, 0.4 % [95 % CI, −7.4 to 8.2 %]; logrank test, *p* = 0.987) (Fig. 2a; Table 2a).

**Table 2:** Disease-free survival rate at the end of 5-year study period

Treatment group	*N*	DFS rate (%)	SE (%)	95 % CI	Difference^b^ (%)	
					DFS rate	95 % CI
**A** Throughout the 5-year study period (240 weeks)^a^						
2 years	112	90.4	2.89	82.9 to 94.7	0.4	−7.4 to 8.2
3 or more years	110	90.8	2.77	83.6 to 94.9		
**B** During the third through fifth year study period (144 weeks after week 96)^c^						
2 years	99	91.8	2.79	84.2 to 95.8	2.3	−4.8 to 9.5
3 or more years	102	94.1	2.34	87.3 to 97.3		

*DFS* disease-free survival, *SE* standard error, *CI* confidence interval, *FAS* full analysis set

^a^DFS for patients in the FAS

^b^Difference = 3-or-more-year treatment group − 2-year treatment group

^c^DFS for patients in the FAS who had an examination at the end of 2-year treatment and continued through the fifth year study period

As the first primary analysis, during the third through fifth year study period, among 201 patients in the mFAS (99 and 102 in the 2- and 3-or-more-year groups, respectively), there were 14 disease events (8 and 6): 5 recurrences (3 and 2) and 9 second primary cancers (5 and 4). The DFS rate at 144 weeks after week 96 was 91.8 % and 94.1 % in the 2- and 3-or-more-year groups, respectively, with no significant between group difference (2.3 % [95 % CI, −4.8 to 9.5 %]). There were no significant differences in DFS between the 2 groups (hazard ratio, 0.739 [95 % CI, 0.257 to 2.131]; logrank test, *p* = 0.575) (Fig. 2b; Table 2b).

For OS throughout the study period, among 222 patients in the FAS, there was 1 death due to breast cancer in the 3-or-more-year group. The OS rate at week 240 was 100 and 99 % in the 2- and 3-or-more-year groups, respectively, with no significant difference between the 2 groups. During the third through fifth year study period, all of the 201 patients in the mFAS survived through the end of the study, with the OS rate of 100 % in both groups at 144 weeks after week 96.

### Serum hormone levels and menstrual status

**Fig. 3:**
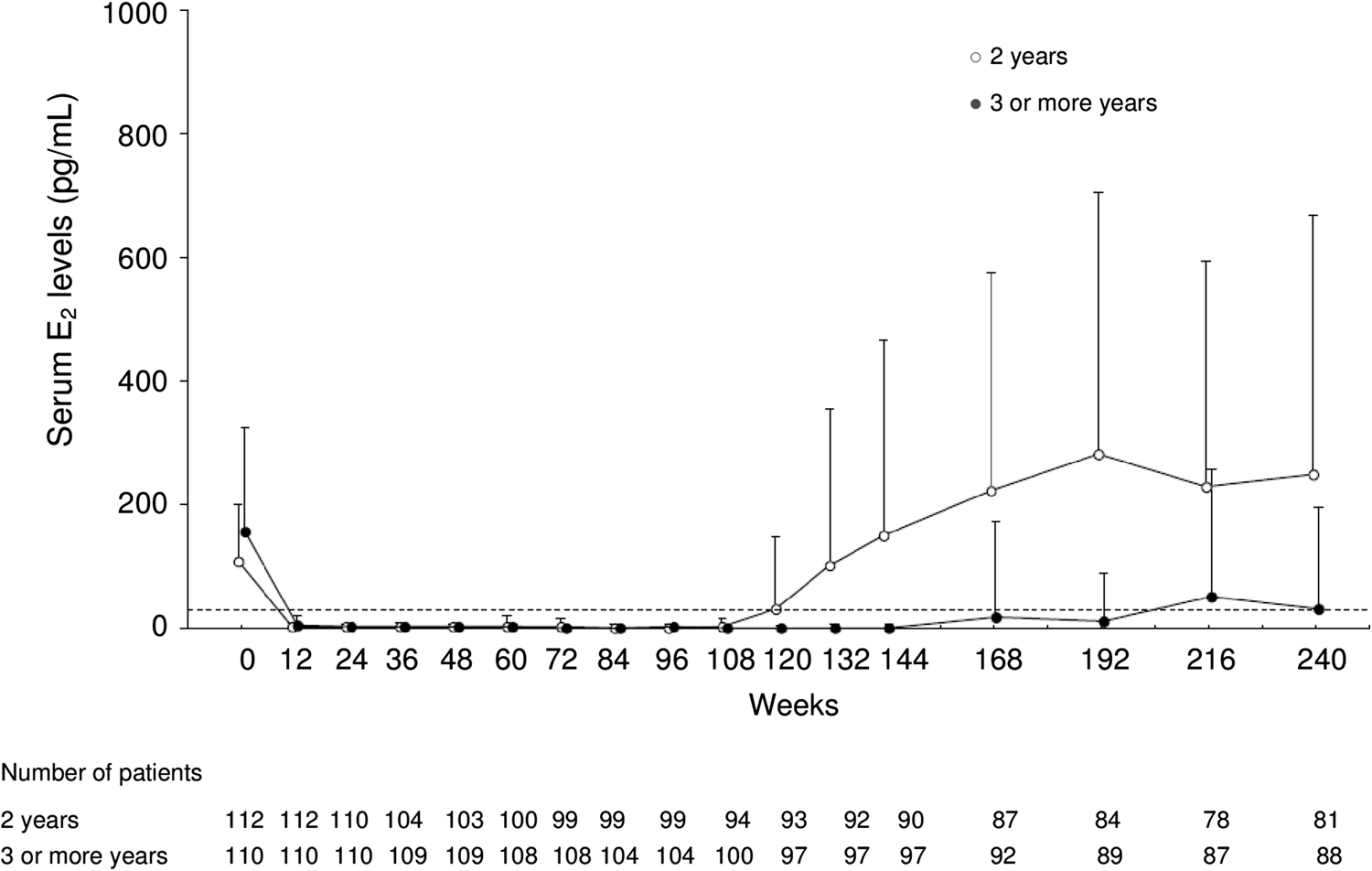
Time course of mean changes from baseline in serum estradiol levels throughout the 5-year study period. Data indicate the mean + SD. *SD* standard deviation, *E*_*2*_ estradiol

Serum levels of E_2_ significantly declined to menopausal levels (<30 pg/mL) after 12 weeks of leuprorelin treatment and remained at the low levels through to the end of its administration in both of the 2 groups (Fig. 3). In the 2-year group, serum E_2_ levels increased gradually after the completion of leuprorelin treatment and recovered to levels almost the same as the pretreatment values by week 132. Similar changes in serum levels of LH and FSH were observed during the overall study period.

Patients achieved amenorrhea during the leuprorelin treatment period, but menses returned in 68 and 19 patients in the 2- and 3-or-more-year groups, respectively, during the follow-up period.

### Safety

Throughout the study period, 96.4 % (108/112) and 98.2 % (108/110) of patients experienced treatment-emergent AEs in the 2- and 3-or-more-year groups, respectively, with no significant difference between the 2 groups. The incidence of treatment-related AEs, however, was significantly higher in the 3-or-more-year group than in the 2-year group (96.4 versus 89.3 %, *p* = 0.041). Table 3 summarizes the most common treatment-related AEs occurring in 10 % or more of patients in each group. The most common treatment-related AEs were hot flush (58.9 and 59.1 % in the 2- and 3-or-more-year groups, respectively), followed by hyperhidrosis (25.0 and 27.3 %), arthralgia (9.8 and 21.8 %), and headache (10.7 and 20.9 %), which were of grades 1 or 2. There were also grade 3 triglyceride increase, uterine polyp and cervical polyp (2 patients each), organizing pneumonia and interstitial lung disease (1 patient each), and grade 4 breast cancer (1 patient) in the 2-year group, and grade 3 gamma-glutamyltransferase increase (4 patients), uterine polyp and cervical polyp (2 patients each), alanine aminotransferase increase, white blood cell count decrease, hypertension, and varicose veins (1 patient each) in the 3-or-more-year group. Serious AEs were found in 12.5 % (14/112) and 11.8 % (13/110) of patients in the 2- and 3-or-more-year groups, respectively. Serious treatment-related AEs included breast cancer, organizing pneumonia, and interstitial lung disease (1 patient each) in the 2-year group, and varicose veins (1 patient) in the 3-or-more-year group.

**Table 3:** Treatment-related adverse events occurring in 10 % or more of patients

System organ class Preferred term (MedDRA version 16.0)	Treatment group	
	2 years (*N* = 112)	3 or more years (*N* = 110)
Any adverse event	100 (89.3)	106 (96.4)
General disorders and administration site conditions		
Injection site induration	19 (17.0)	20 (18.2)
Hepatobiliary disorders		
Hepatic steatosis	11 (9.8)	15 (13.6)
Investigations		
Blood triglycerides increased	9 (8.0)	15 (13.6)
Gamma-glutamyltransferase increased	8 (7.1)	14 (12.7)
Bone density decreased	7 (6.3)	11 (10.0)
Musculoskeletal and connective tissue disorders		
Arthralgia	11 (9.8)	24 (21.8)
Musculoskeletal stiffness	13 (11.6)	12 (10.9)
Nervous system disorders		
Headache	12 (10.7)	23 (20.9)
Dizziness	12 (10.7)	8 (7.3)
Psychiatric disorders		
Insomnia	12 (10.7)	7 (6.4)
Reproductive system and breast disorders		
Metrorrhagia	13 (11.6)	7 (6.4)
Skin and subcutaneous tissue disorders		
Hyperhidrosis	28 (25.0)	30 (27.3)
Vascular disorders		
Hot flush	66 (58.9)	65 (59.1)

Values represent the number (%) of patients

*MedDRA* medical dictionary for regulatory activities

In total, 6 patients discontinued leuprorelin treatment due to the treatment-related AEs (2 patients with organizing pneumonia, 1 each with extremity pain, depression, genital hemorrhage, or interstitial lung disease) in the 2-year group; and 5 patients discontinued leuprorelin treatment due to the treatment-related AEs (2 patients with depression, 1 patient with depressive symptoms, edema, musculoskeletal stiffness and hot flush, and 1 patient each with injection site induration, or altered mood) in the 3-or-more-year group.

The incidence of treatment-emergent AEs, which occurred during the third through fifth year study period was 83.8 % (83/99) and 87.3 % (89/102) in the 2- and 3-or-more-year groups, respectively, with no significant difference between the 2 groups. Most treatment-related AEs were of grade 1 and 2, but grade 3 uterine polyp (2 patients), cervical polyp (1 patient), and grade 4 breast cancer (1 patient) were observed in the 2-year group, and grade 3 alanine aminotransferase increase and hypertension (1 patient each) were reported in the 3-or-more-year group. Serious treatment-related breast cancer was observed in the 2-year group. Only 1 patient in the 3-or-more-year group discontinued leuprorelin treatment because of treatment-related non-serious musculoskeletal stiffness.

### Bone mineral density

**Fig. 4:**
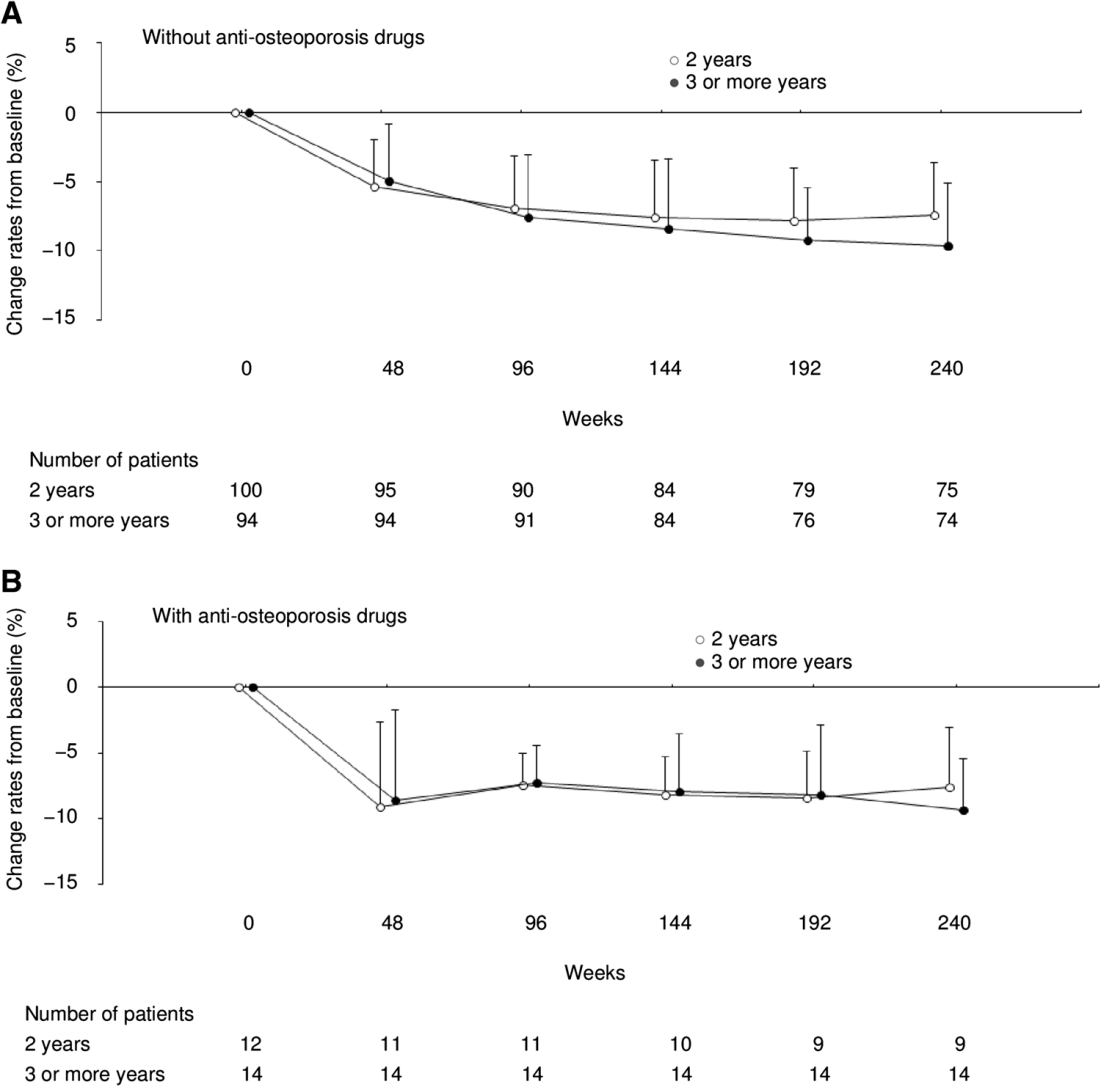
Time course of the mean change rates from baseline in bone mineral density in the lumbar spine in patients **a** without or **b** with concomitant anti-osteoporosis treatment throughout the 5-year study period. Data indicate the mean + SD. *SD* standard deviation

Figure 4 shows the time course of mean change rates from baseline in BMD in the lumbar spine. For patients who did not receive concomitant administration of anti-osteoporosis drugs, the mean change rates in BMD at weeks 192 and 240 were −7.871 % (95 % CI, −8.7339 to −7.0072) and −7.416 % (95 % CI, −8.2879 to −6.5443) in the 2-year group, and −9.267 % (95 % CI, −10.1444 to −8.3906) and −9.682 % (95 % CI, −10.7400 to −8.6238) in the 3-or-more-year group, respectively. The reduction of BMD was significantly greater at both assessment time points in the 3-or-more-year group than in the 2-year group, which had completed leuprorelin treatment at week 96. For patients receiving concomitant anti-osteoporosis drugs, there were no significant differences in the mean change rates in BMD between the 2 groups throughout the study period.

Throughout the study period, the incidence of bone-related AEs in patients without anti-osteoporosis drugs was 6.0 % (6/100) and 6.3 % (6/96) in the 2- and 3-or-more-year groups, respectively, and that in patients with anti-osteoporosis drugs was 66.7 % (8/12) and 78.6 % (11/14) in the 2- and 3-or-more-year groups, respectively.

During the third through fifth year study period, the incidence of bone-related AEs in patients without anti-osteoporosis drugs was 2.3 % (2/88) and 4.5 % (4/88) in the 2- and 3-or-more-year groups, respectively, and that in patients with anti-osteoporosis drugs was 18.2 % (2/11) and 28.6 % (4/14) in the 2- and 3-or-more-year groups, respectively. It was noted that all of the bone-related AEs observed in patients with anti-osteoporosis drugs occurred before the initiation of osteoporosis treatment.

## Discussion

Our study suggests that adjuvant leuprorelin treatment for 3 or more up to 5 years with tamoxifen for 5 years resulted in a little higher DFS rate at week 240 compared with 2 years of leuprorelin treatment with tamoxifen, in particular, during the third through fifth year study period; nevertheless, there were no significant differences between the 2 groups (Table 2; Fig. 2). Moreover, the OS rate was 100 % in both of the groups during the third through fifth year study period. Although the number of patients in this study was insufficient to clarify the difference between the DFS rates in the 2 groups, only 10 disease events each were found and good efficacy was shown in the 2 groups throughout the 5-year study period. Although hormone receptor-positive breast cancer has a relatively good prognosis, the risk of recurrence remains at 5 years or more after surgery. Therefore, a longer follow-up, such as observation for 10 years will be necessary to evaluate the optimal treatment duration for postoperative adjuvant tamoxifen plus LH-RH therapy.

After the completion of leuprorelin treatment, serum E_2_ levels increased gradually and recovered to the pretreatment levels (Fig. 3). Menses resumed in 68 and 19 patients in the 2- and 3-or-more-year groups, respectively, during the follow-up period. The number of patients with resumption of menses in the 3-or-more-year group was less than that in the 2-year group, because about 70 % of 110 patients in the 3-or-more-year group received 5 years of leuprorelin treatment, which resulted in a shorter follow-up period after the end of treatment than that for the 2-year group.

Although the incidence of treatment-related AEs was significantly higher in the 3-or-more-year group than in the 2-year group (Table 3), the majority of treatment-related AEs were of grades 1 and 2, and were considered to be associated with the pharmacological activity of leuprorelin. The increase in the treatment duration of leuprorelin led to a decrease in the BMD, but there were neither increases in the severity of AEs nor occurrences of any new types of AEs. The safety profile of the 3 or more years of leuprorelin treatment is comparable to that of the 2 years of its treatment. No new safety signal was identified for long-term treatment with leuprorelin. These findings thus demonstrated that adjuvant leuprorelin treatment for 3 or more up to 5 years concomitant with tamoxifen for 5 years was safe and well tolerated.

For patients without concomitant anti-osteoporosis drugs, leuprorelin treatment for 3 or more years led to a significantly greater reduction in the BMD in the lumbar spine at weeks 192 and 240 compared with its treatment for 2 years (Fig. 4a). For patients with anti-osteoporosis drugs, however, there were no significant differences in the mean change rates in BMD between the 2 groups throughout the study period (Fig. 4b). This suggests that the concomitant use of anti-osteoporosis drugs may be the preferred choice for premenopausal women with endocrine-responsive breast cancer to manage and mitigate the potential osteoporosis condition associated with long-lasting adjuvant ovarian suppression therapy. In line with this notion, the ABCSG-12 study reported that addition of the bisphosphonate drug zoledronic acid to 3 years of adjuvant endocrine therapy of goserelin plus tamoxifen resulted in an increase in the BMD of the lumbar spine beyond the pretreatment levels in premenopausal women with endocrine-responsive early breast cancer [22]. In the present study, the mean change rate in the BMD from baseline was −8.4 % at week 144 in the 3-or-more-year group for patients without anti-osteoporosis drugs, which is similar to that (−9.0 %) reported in patients who received goserelin plus tamoxifen therapy in the ABCSG-12 study. Although it is difficult to directly compare the present results with those reported in the ABCSG-12 study, since in the present study, all types of anti-osteoporosis drugs were available as needed and a variety of treatment durations and timing were used, our present results indicate that the concomitant use of anti-osteoporosis drugs may prevent the bone loss associated with leuprorelin treatment.

The optimal duration of adjuvant ovarian suppression therapy with an LH-RH agonist alone or in combination with adjuvant tamoxifen or chemotherapy in premenopausal women with endocrine-responsive breast cancer has been one of the most controversial issues [23]. Very recently, an important result of the Suppression of Ovarian Function Trial (SOFT), which investigated the efficacy of 5-year administration of tamoxifen alone or in combination with 5-year ovarian ablation with an LH-RH agonist, was reported. The trial showed that ovarian suppression in addition to tamoxifen did not provide a significant benefit to the overall study population [24]. However, in premenopausal patients who were at high risk for recurrence and were treated with adjuvant chemotherapy, the combination of an LH-RH agonist and tamoxifen was reported to reduce recurrence and death compared with tamoxifen alone [24]. Thus, it is expected that an increasing number of premenopausal patients at high risk for recurrence will in the future be treated with a combination of an LH-RH agonist and tamoxifen for 5 years. In this context, our present study is important because it provides valuable information on the safety of long-term administration of an LH-RH agonist plus tamoxifen for up to 5 years, which may help to make a decision on the indication for 5-year administration of an LH-RH agonist plus tamoxifen to Japanese breast cancer patients who were not studied in SOFT.

Taking the feasibility in Japan only into consideration, this study was planned as a pilot study. In addition to longer follow-up observation, further clinical trials with larger patient populations will be needed to evaluate the utility of the long-term postoperative adjuvant endocrine therapy of leuprorelin plus tamoxifen, and to determine the optimal duration of leuprorelin treatment for premenopausal women with endocrine-responsive breast cancer.
